# DNA Barcodes Perform Best with Well-Characterized Taxa

**DOI:** 10.1371/journal.pbio.0030435

**Published:** 2005-11-29

## Abstract

.

With species around the world disappearing faster than biologists can identify them, the need for rapid, accurate methods of classifying life has never been more pressing. Toward this end, many scientists pinned their hopes on DNA barcoding, a recently proposed strategy that treats a short fragment of DNA as a sort of universal product code to identify species by running unknown sequences through a database that links DNA barcodes to organisms. But this approach generated controversy from the start, with advocates touting the benefits—rapid identification of unknown individuals and discovery of novel species—and skeptics bristling at the notion that a single gene fragment could perform such a tall task.

For most animals, the DNA barcode consists of just over 600 base pairs of a mitochondrial gene called *cytochrome oxidase subunit I (COI)*. In September 2004, *PLoS Biology* published a paper that tested *COI* barcode performance using a proportion of North American birds. The study found that all these well-studied species had a different barcode, and that the variation between species was much higher than variation within species. Based on this gap, the study proposed a screening threshold of sequence difference (ten times the average within-species difference) that could speed the discovery of new animal species. In a new study, Christopher Meyer and Gustav Paulay revisit the issue with a diverse, extensively studied snail group, the ubiquitous, tropical marine cowries whose shell can command over $30,000. Meyer and Paulay found that while the barcode worked well for identifying specimens in highly characterized groups, thresholds would miss many novel species.

After ten years of collecting and sequencing cowries from around the world, Meyer and Paulay assembled a database of over 2,000 cowrie *COI* sequences from 218 species. To capture the full range of within-species variation and geographic differences in population structure, they included sequences from multiple individuals and geographic extremes. Meyer and Paulay tested barcode performance in species identification and discovery against traditional morphology-based species lists and against an integrated taxonomic approach that determines “evolutionary significant units” (ESUs) based on morphology and sequence data. ESUs are what's called reciprocally monophyletic—two ESUs each have a unique ancestor, and, therefore, a unique genetic signature. But genetic variation doesn't always track with species distinctions. The common ancestor of some species nests within another species' variation (called paraphyly), and sometimes different members of what is thought to be one species can be related to another species and not share a most recent common ancestor (called polyphyly). [Fig pbio-0030435-g001]

**Figure pbio-0030435-g001:**
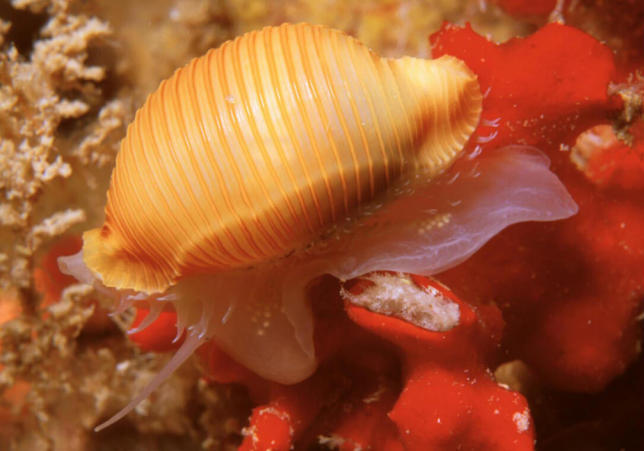
The charismatic cowrie

Meyer and Paulay found that barcodes could accurately identify unknown samples against a well-characterized database using ESUs, but were “prone to error”—with a 20% failure rate—when traditional species checklists were used, likely reflecting taxonomic problems mentioned above (lumping similar forms that turn out to be distinct species, for example, or erroneously classifying a specimen with an odd morphology as separate species).

When Meyer and Paulay looked at thresholds to delineate species in cowries, they found considerable abundance of young taxa between intra- and intertaxon variation at both ESU and species levels. Within-species variation among cowries was “substantially higher” than that found in two other marine snails, limpets and turbinids, demonstrating the value of comparative analyses in generalizing limits for intra-species variation. The three groups also showed a wide range of interspecies variation. Still, using a barcode threshold to constrain intraspecies variation worked well for ESUs (98% of the taxa had less than 3% variation).

But error rates were substantial when applying thresholds to species discovery because of the abundance of young taxa. For instance, of the 263 ESUs, 16% artificially lumped with another ESU at the 3% threshold; similar patterns were seen in the turbinids and limpets. Because many traditionally recognized cowrie species are not reciprocally monophyletic based on their *COI* barcode, when Meyer and Paulay replaced cowrie ESUs with recognized species, both intra- and interspecies variation increased, bumping the error rate above 30%.

This comprehensive analysis demonstrates that relying solely on DNA barcodes masks fine-tuned species boundaries not readily captured in DNA sequences without extensive sampling. The barcode performs best in identifying individuals against a well-annotated sequence database—as demonstrated here with ESUs—and the authors argue that the barcoding movement is well-equipped to help in this effort. But barcoding methods for discovering new species need refinement, they argue, and should be developed in collaboration with taxonomists, systematists, and ecologists into a comprehensive taxonomic framework. Once databases are fully annotated with taxonomically evaluated sequences, error rates should go down. With just 1.7 million species described and some 10 million to go, there's a lot of work to be done. —*Liza Gross*

